# How Cholesterol Constrains Glycolipid Conformation for Optimal Recognition of Alzheimer's β Amyloid Peptide (Aβ_1-40_)

**DOI:** 10.1371/journal.pone.0009079

**Published:** 2010-02-05

**Authors:** Nouara Yahi, Anaïs Aulas, Jacques Fantini

**Affiliations:** Université Paul Cézanne (Aix-Marseille 3), Université de la Méditerranée (Aix-Marseille 2), Centre de Recherche en Neurobiologie et Neurophysiologie de Marseille, CNRS UMR 6231, INRA USC 2027, Interactions Moléculaires et Systèmes Membranaires, Faculté des Sciences Saint-Jérôme, Marseille, France; University of Nebraska Medical Center, United States of America

## Abstract

Membrane lipids play a pivotal role in the pathogenesis of Alzheimer's disease, which is associated with conformational changes, oligomerization and/or aggregation of Alzheimer's β-amyloid (Aβ) peptides. Yet conflicting data have been reported on the respective effect of cholesterol and glycosphingolipids (GSLs) on the supramolecular assembly of Aβ peptides. The aim of the present study was to unravel the molecular mechanisms by which cholesterol modulates the interaction between Aβ_1–40_ and chemically defined GSLs (GalCer, LacCer, GM1, GM3). Using the Langmuir monolayer technique, we show that Aβ_1–40_ selectively binds to GSLs containing a 2-OH group in the acyl chain of the ceramide backbone (HFA-GSLs). In contrast, Aβ_1–40_ did not interact with GSLs containing a nonhydroxylated fatty acid (NFA-GSLs). Cholesterol inhibited the interaction of Aβ_1–40_ with HFA-GSLs, through dilution of the GSL in the monolayer, but rendered the initially inactive NFA-GSLs competent for Aβ_1–40_ binding. Both crystallographic data and molecular dynamics simulations suggested that the active conformation of HFA-GSL involves a H-bond network that restricts the orientation of the sugar group of GSLs in a parallel orientation with respect to the membrane. This particular conformation is stabilized by the 2-OH group of the GSL. Correspondingly, the interaction of Aβ_1–40_ with HFA-GSLs is strongly inhibited by NaF, an efficient competitor of H-bond formation. For NFA-GSLs, this is the OH group of cholesterol that constrains the glycolipid to adopt the active L-shape conformation compatible with sugar-aromatic CH-π stacking interactions involving residue Y10 of Aβ_1–40_. We conclude that cholesterol can either inhibit or facilitate membrane-Aβ interactions through fine tuning of glycosphingolipid conformation. These data shed some light on the complex molecular interplay between cell surface GSLs, cholesterol and Aβ peptides, and on the influence of this molecular ballet on Aβ-membrane interactions.

## Introduction

Alzheimer's disease is a neurodegenerative pathology of the central nervous system currently affecting more than 25 millions of individuals worldwide. It is characterized by the presence of neuritic plaques and neurofibrillary tangles which contribute to neuronal and synaptic loss. Although the molecular and cellular mechanisms responsible for Alzheimer's disease are not fully understood, the formation of insoluble deposit of the β-amyloid peptide (Aβ) fragments seems to play a major role in the pathogenesis of the disease [1], [2]. What we call Aβ is in fact a family of peptides derived by the proteolytic cleavage of the amyloid precursor protein (APP), a transmembrane protein expressed in neuronal but also in non-neuronal tissue [3]. The most abundant forms of Aβ are 40- and 42-amino acid peptides respectively referred to as Aβ_1–40_ and Aβ_1–42_. Both peptides are found in amyloid plaques that, according to the amyloid cascade hypothesis [2], [4], eventually lead to the neurodegeneration. The aggregation process of amyloid peptides is driven by the supramolecular assembly of beta sheet structures. Non-fibrillar oligomers of Aβ are also toxic [5], and it is difficult to assess which molecular species among dimers, oligomers and fibrils are the most pathogenic.

Given that these amyloid peptides are released in the immediate vicinity of the plasma membrane in which APP is anchored, it is not surprising that several membrane lipids can interact with Aβ and affect the oligomerization process [6]. Lipid rafts, which are membrane microdomains enriched in cholesterol and sphingolipids [7], have been proposed to act as surface catalysts able to accelerate the aggregation of Aβ [8]. Aβ peptides interact with several glycosphingolipids (GSLs), including both neutral GSLs such as asialo-GM1 [9] or galactosylceramide (GalCer) [10], [11] and gangliosides such as GM1 [12]. Conflicting data have suggested that binding to GM1 induces either a conformational transition from random coil to a protective α-helical structure [13] or to a fibrillation-prone β-structure [14], [15]. As a matter of fact, the structure of Aβ bound to GM1 depends on several physicochemical parameters including pH, membrane fluidity, GM1-carrier lipid ratios, Aβ concentration, and the presence of cholesterol, which could explain the discrepancies reported in the literature.

Although cholesterol has been identified as a major risk factor for Alzheimer's disease [16], its effects on Aβ fibrillogenesis and toxicity are not well understood and the results reported so far are controversial [17]. Cholesterol stimulates the insertion of APP into phospholipid monolayers [18] and it binds to Aβ_1–42_ protofibrils [19]. However, whether cholesterol accelerates [20] or decreases [21], [22] Aβ polymerization is still uncertain. Moreover, the generation of Aβ peptides through APP proteolysis occurs within lipid rafts and is sensitive to inhibitors of cholesterol biosynthesis [16], so that the involvement of cholesterol homeostasis in Alzheimer's disease cannot be simply ascribed to the regulation of Aβ fibrillogenesis.

There is one important issue that has not been addressed in these investigations. GSLs exhibit a high biochemical diversity in both their glycone and ceramide moieties [23]. Perhaps the most widely neglected biochemical characteristic of GSLs in amyloid studies is the existence of two distinct types of ceramide backbones which are defined by the absence or presence of an OH group bound to the C2 of the fatty acid chain [24]. Thus, major brain GSLs such as GalCer actually exist as 2-hydroxy (2-OH) fatty acid (HFA) or non-hydroxy fatty acid (NFA) species, in roughly equivalent amounts [25]. The hydroxylation status of the ceramide has a major impact on the conformation of the GSL, as elegantly demonstrated by I. Pascher and co-workers for GalCer [26], [27]. An intramolecular H-bond network in GalCer-HFA stabilizes the parallel orientation of the galactose ring with respect to the lipid membrane, giving the molecule a typical L-shape [27]. In contrast, the lack of this OH group in GalCer-NFA allows the galactose head group to freely adopt an extended conformation in a layer-perpendicular position. Proteins that recognize the L-shape conformation of GalCer-HFA can totally ignore GalCer-NFA, as perfectly demonstrated for the HIV-1 surface envelope glycoprotein gp120 [28].

Here we studied the interaction of Aβ_1–40_ with various HFA and NFA GSLs and the effects of cholesterol on these interactions. We show that Aβ_1–40_ has a marked preference for HFA vs. NFA GSLs. This effect was reproducibly observed with both natural and synthetic GSLs with defined fatty acid content. Cholesterol was found to inhibit the interaction of Aβ_1–40_ with the HFA species but to strongly stimulate the interaction with NFA GSLs. Using a combination of physicochemical and molecular modeling approaches, we show that the 2-OH group of the fatty acid and the OH group of cholesterol have a similar conformational effect on various GSLs.

## Materials and Methods

### Materials

Natural GalCer-NFA (kerasin), GalCer-HFA (phrenosin), GM1 and GM3 were obtained from Matreya (Pleasant Gap, PA). Synthetic GalCer with C12:0 fatty acid chain (GalCer-C12) and LacCer with C8:0 fatty acid chain (LacCer-C8) were purchased from Avanti Polar Lipids (Alabaster, AL). Synthetic cholesterol and phosphatidylserine were from Sigma (Saint-Quentin Fallavier, France). Synthetic Aβ_1–40_ was from Sigma. All lipids were dissolved at a concentration of 1 mg.mL^−1^ in hexane:chloroform:ethanol (11∶5∶4, vol:vol:vol). Sterile ultrapure water (endotoxin ≤0.25 EU.mL^−1^; conductivity 1.1 µS.cm^−1^ at 20°C; resistivity 0.91 MΩ.cm at 20°C; surface tension 72.8 mN.m^−1^; pH 6.9) conditioned in 9 mL tubes was obtained from Biorad (Marnes-La-Coquette, France). All other reagents, of the highest quality available, were from Sigma.

### Surface Pressure Measurements

Surface pressure measurements revealing peptide-lipid interactions were studied by the Langmuir film balance technique with a fully automated microtensiometer (µTROUGH SX, Kibron Inc. Helsinki, Finland). Indeed, the interaction of a peptide (or a protein) with a glycolipid monolayer is an interfacial phenomenon which can be studied by surface pressure (π) measurements [6]. Namely, the insertion of the peptide in the lipid monolayer can be detected, at constant area, by an increase in the surface pressure (Δπ). This increase in the surface pressure is caused by the insertion of the peptide between the polar heads of vicinal glycolipids in the monolayer, which is not counterbalanced by an increase of the area of the monolayer. This effect can be followed kinetically by real-time surface pressure measurements after injecting the peptide into the aqueous subphase underneath the lipid monolayer as described previously [29]. Such [Δπ  =  f(t)] curves allow to assess the actual interaction of a peptide (or a soluble protein) with a given glycolipid. The initial velocity (v_i_) of the insertion process is expressed as mN.m^−1^.min^−1^. The difference between the maximal (π_max_) and the initial (π_i_) surface pressure values allows to calculate the maximal surface pressure increase (Δπ_max_) induced by the peptide (expressed in mN.m^−1^). Mixed monolayers were prepared from stock solutions of lipid mixtures (1∶1, mol:mol). Unless otherwise indicated, the subphase underneath the lipid monolayer is ultrapure water (pH 6.9 for all experiments). In any case, the subphase was prepared extemporaneously from disposable sterile units. All experiments were conducted at 20°C in triplicate. Results were expressed as means ± standard deviation (S.D.).

### Molecular Modeling Simulations

The structure of Aβ_1–40_ has been retrieved from the PDB entry # 1BA4 [30]. The structure of GSLs were derived from those published by Pascher & Sundell [26]. The whole structure of Aβ_1–40_ was merged with GalCer-HFA or GalCer-NFA + cholesterol. Molecular dynamics studies of GSLs and Aβ-GSL interactions were performed with the Hyperchem program, using with the MM+ force field as described previously [29], [31]. The Polak-Ribiere algorithm was used to calculate the minimal energy of the individual molecules (GSLs and peptides) in each system. Molecular dynamics simulations were then performed for 1 ps. The introduction of water molecules in the periodic box did not influence the establishment of sugar-aromatic stacking interactions [29]. Indeed, similar data were obtained when the modeling of this interaction was performed in vacuo and in water. To improve clarity, water molecules were not represented in the molecular models.

### Chemical Structures

All chemical structures were drawn with ChemDraw.

## Results

### Effect of Cholesterol on the Interaction between GalCer and Aβ_1–40_

**Figure 1:**
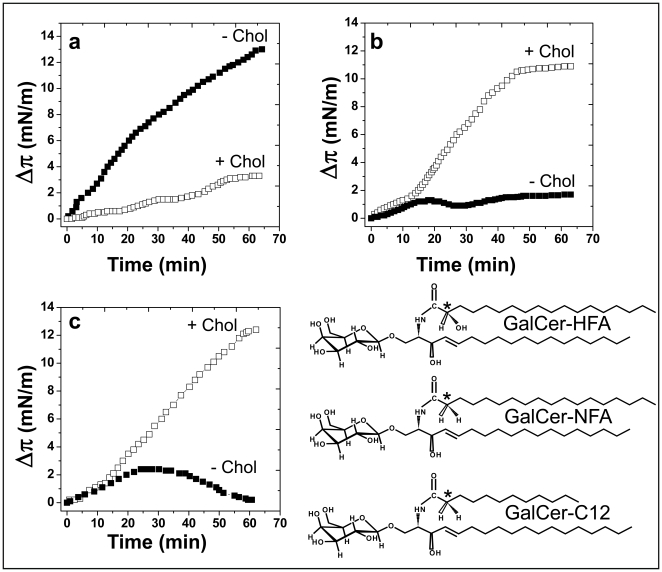
Effects of cholesterol on the recognition of GalCer-HFA and GalCer-NFA by Aβ_1–40_. **a–c**. Kinetics of Aβ_1–40_ insertion into a monolayer of natural GalCer-HFA (**a**), natural GalCer-NFA (**b**) or GalCer-C12 (**c**) in either the absence (▪) or presence of cholesterol (□). The pure GalCer and mixed GalCer/cholesterol (1∶1, mol:mol) monolayers were prepared at an initial surface pressure of 13–15 mN.m^−1^ as indicated in Materials and Methods. The data show the evolution of the surface pressure following the injection of Aβ_1–40_ (1 µM) in the aqueous phase underneath the monolayer. Each experiment was performed in triplicate and one representative curve is shown (S.D. <10%). The chemical structures of GalCer-HFA (with a C18:0 2-OH fatty acid), GalCer-NFA (with a C18:0 fatty acid) and GalCer-C12 (with a C12:0 fatty acid) are shown in the lower right panel. The stereochemistry of the C2 atom is indicated by an asterisk.

In a first set of experiments we investigated the interaction of Aβ_1–40_ with GalCer-HFA (**Figure 1a**) and GalCer-NFA (**Figure 1b**) purified from bovine brain (see **Table 1** for a description of the fatty acid content of the GSLs used in the present study, and **Figure 1** for GalCer structures). We used the Langmuir monolayer technique which has been widely validated for lipid-protein studies and has proven particularly useful for reconstituting sphingolipid-cholesterol systems (for review, see [6]). Monolayers of these GSLs were prepared at the air-water interface on a subphase of ultrapure water (the use of water subphases for such surface pressure studies has been extensively characterized and validated in previous studies [28], [29], [31]). The peptide was injected in the subphase and the surface pressure was continuously measured with a microtensiometer. As shown in **Figure 1a**, the surface pressure started to increase immediately after the injection of the peptide underneath the GalCer-HFA monolayer, with an initial velocity (v_i_) of 0.266±0.023 mN.m^−1^.min^−1^ (n = 3, **Table 2**). A maximal value of 12.2±0.9 mN.m^−1^ (n = 3) was reached after 65 min of interaction (Δπ_max_). In contrast, the peptide interacted very weakly with a monolayer of GalCer-NFA (**Figure 1b**). Following an initial slow increase (v_i_  = 0.033±0.004 mN.m^−1^.min^−1^ (n = 3, **Table 2**), the surface pressure peaked after 18 min of interaction (Δπ_max_  = 1.5±0.1 mN.m^−1^, n = 3, **Table 2**). This indicates that Aβ_1–40_ has a high affinity for GalCer-HFA and a very low affinity for GalCer-NFA. The presence of cholesterol in these monolayers (i.e. mixed GalCer/cholesterol monolayers) had opposite effects for both GalCer species. In the case of GalCer-HFA (**Figure 1a**), cholesterol markedly inhibited the interaction of Aβ_1–40_ with the monolayer (v_i_ = 0.031±0.004 mN.m^−1^.min^−1^; Δπ_max_  = 3.2±0.2 mN.m^−1^, n = 3, **Table 2**). Compared with pure monolayers of GalCer-HFA, this corresponds to a 8.1-fold decrease in v_i_ and a 3.8-fold decrease in Δπ_max_. In contrast, cholesterol markedly stimulated the interaction of Aβ_1–40_ with GalCer-NFA (**Figure 1b**). During the first 15 min following the injection of the peptide underneath a mixed GalCer-NFA/cholesterol monolayer, the interaction occurred with a v_i_ = 0.107±0.008 mN.m^−1^.min^−1^ (n = 3, **Table 2**). Then the velocity of the interaction further increased to reach 0.308±0.027 mN.m^−1^.min^−1^ (n = 3). A Δπ_max_ of 10.4 mN.m^−1^±0.6 (n = 3, **Table 2**) was reached after 47 min of incubation, corresponding to a 6.9-fold increase. That the interaction proceeded as a two-step phenomenon with an initial slow velocity followed by a more rapid one suggests that a conformational adjustment is required for an optimal interaction.

**Table 1:** Typical fatty acid composition of the natural glycosphingolipids used in this study*.

Fatty Acids	GalCer-NFA	GalCer-HFA	GM3	GM1
**C16:0**	–	–	6	1
**C18:0**	5	–	1	86
**C18:1**	–	–	–	3
**C20:0**	1	–	1	4
**C21:0**	–	–	1	–
**C22:0**	9	–	23	2
**C23:0**	5	–	36	1
**C24:0**	25	–	22	1
**C24:1**	43	–	3	2
**C25:0**	3	–	–	–
**C25:1**	3	–	–	–
**C26:0**	2	–	–	–
**C26:1**	4	–	–	–
**C18:0 2-OH**	–	36	–	–
**C20:0 2-OH**	–	1	–	–
**C22:0 2-OH**	–	8	–	–
**C23:0 2-OH**	–	6	–	–
**C24:0 2-OH**	–	25	–	–
**C24:1 2-OH**	–	9	–	–
**C25:0 2-OH**	–	4	–	–
**C25:1 2-OH**	–	2	–	–
**C26:0 2-OH**	–	2	–	–
**C26:1 2-OH**	–	2	–	–
**Others**	–	5	7	–
**Total**	100	100	100	100

*according to the supplier (Matreya, Pleasant Gap, PA).

**Table 2:** Physicochemical parameters of the interaction between Aβ_1–40_ and the indicated lipid monolayers.

Lipid	V_i_ (mN.m^−1^.min^−1^)	Δπ_max_ (mN.m^−1^)
GalCer-HFA	0.266±0.023	12.2±0.9
GalCer-HFA:cholesterol (1∶1; mol:mol)	0.031±0.004	3.2±0.2
GalCer-NFA	0.033±0.004	1.5±0.1
GalCer-NFA:cholesterol (1∶1; mol:mol)	0.107±0.008	10.4±0.6
GalCer-C12	0.091±0.008	2.1±0.1
GalCer-C12:cholesterol (1∶1, mol:mol)	0.117±0.009	12.2 ±1.2
Cholesterol	0.000±0.000	1.0±0.1
PS:cholesterol (1∶1, mol:mol)	0.000±0.000	1.4±0.1
LacCer-C8	0.114±0.009	2.6±0.2
LacCer-C8:cholesterol (1∶1, mol:mol)	0.335±0.028	16.7±1.2
GM3	0.169±0.013	4.1 ±0.3
GM3:cholesterol (1∶1, mol:mol)	0.254±0.019	10.1±0.7
GM1	0.085±0.007	6.3±0.6
GM1:cholesterol (1∶1, mol:mol)	0.161±0.014	9.8±0.7
GalCer-HFA subphase NaCl 0.1 M	0.555±0.046	18.4±1.3
GalCer-HFA subphase NaCl 1 M	0.822±0.077	21.3±1.8
GalCer-HFA subphase NaF 0.1 M	0.078±0.005	3.1±0.2
GalCer-HFA subphase NaF 1 M	0.000±0.00	0.0±0.0

Each monolayer was prepared at initial pressure (π_i_) of 13–15 mN.m^−1^.

The synthetic Aβ_1–40_ peptide was injected in the subphase which consisted of pure water or, when indicated, of NaCl or NaF solutions. Each experiment was performed in triplicate. The data are expressed as the mean ± SD (n = 3). An initial v_i_ with a null value means that there is no increase in the surface pressure over the 20 first minutes of incubation.

The data obtained with natural GalCer-NFA purified from bovine brain were fully confirmed with synthetic GalCer-NFA, i.e. GalCer with a C12:0 nonhydroxylated fatty acid (**Figure 1c**). Indeed, in absence of cholesterol, the surface pressure moderately increased after the injection of Aβ_1–40_ (v_i_ = 0.091±0.008 mN.m^−1^.min^−1^, n = 3, **Table 2**), then reached a plateau at 2.1±0.1 mN.m^−1^ (n = 3, **Table 2**) after 20 min and finally decreased to null values after 60 min of incubation. This decrease could be due to the secondary release of the small amounts of peptide initially adsorbed onto the monolayer, consistent with the low affinity of Aβ for GalCer-C12. As for natural GalCer-NFA, cholesterol stimulated the interaction between Aβ and synthetic GalCer-C12 (v_i_ = 0.117±0.009 mN.m^−1^.min^−1^; Δπ_max_  = 12.2±1.2 mN.m^−1^, n = 3, **Table 2**). This corresponded to a strong stimulation of Δπ_max_ (×5.8 times) with only a minor effect on v_i_ (×1.28 times), in perfect agreement with the data obtained with natural GalCer-NFA. Indeed, as for GalCer-NFA, the interaction followed a two-step kinetic with an initial slow velocity (**Table 2**) followed by a more rapid one (0.203±0.014 mN.m^−1^.min^−1^, n = 3, **Figure 1c**).

**Figure 2 pone-0009079-g002:**
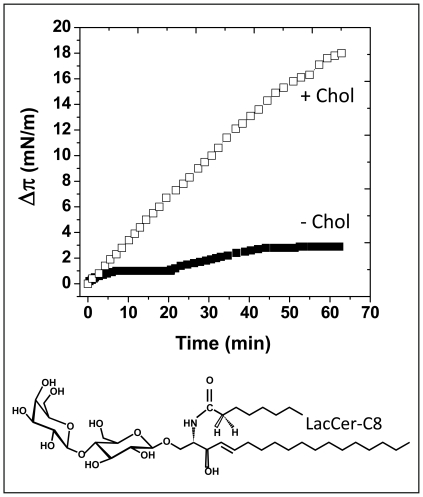
Effects of cholesterol on the recognition of synthetic LacCer (LacCer-C8) by Aβ_1–40_. Upper panel: the kinetics of Aβ_1–40_ insertion into a monolayer of synthetic LacCer-C8 (with a C8:0 fatty acid) were measured in either the absence (▪) or presence of cholesterol (□). The pure LacCer and mixed LacCer/cholesterol (1∶1, mol:mol) monolayers were prepared at an initial surface pressure of 13–15 mN.m^−1^ as indicated in Materials and Methods. The data show the evolution of the surface pressure following the injection of Aβ_1–40_ (1 µM) in the aqueous phase underneath the monolayer. Each experiment was performed in triplicate and one representative curve is shown (S.D. <10%). The chemical structure of LacCer-C8 is shown in the lower panel.

Several control experiments were conducted with various compositions of the lipid monolayer (**Table 2**). Firstly, we demonstrated that cholesterol by itself, at the surface pressure used in the experiments described in **Figure 1** (i.e. 13–15 mN.m^−1^), did not interact with Aβ_1–40_. Then we prepared a mixed monolayer with cholesterol and an irrelevant lipid (phosphatidylserine, PS) to ensure that the stimulating effect of cholesterol on GalCer-NFA was specific. This was indeed the case since in the same surface pressure range (13–15 mN.m^−1^), Aβ_1–40_ did not significantly interact with PS-cholesterol monolayers. Finally, cholesterol could also stimulate the interaction between Aβ_1–40_ and LacCer-C8, a synthetic form of Galβ1-4Glcβ1-Cer with a C8:0 fatty acid (Figure 2). As for GalCer-NFA and GalCer-C12, Aβ_1–40_ showed very little interaction with a monolayer of pure LacCer-C8. The presence of cholesterol in the monolayer triggered the insertion of Aβ, as demonstrated by the increase in both v_i_ (×2.93) and Δπ_max_ (×6.42) (**Table 2**).

### Effect of Cholesterol on the Interaction between Gangliosides and Aβ_1–40_

**Figure 3:**
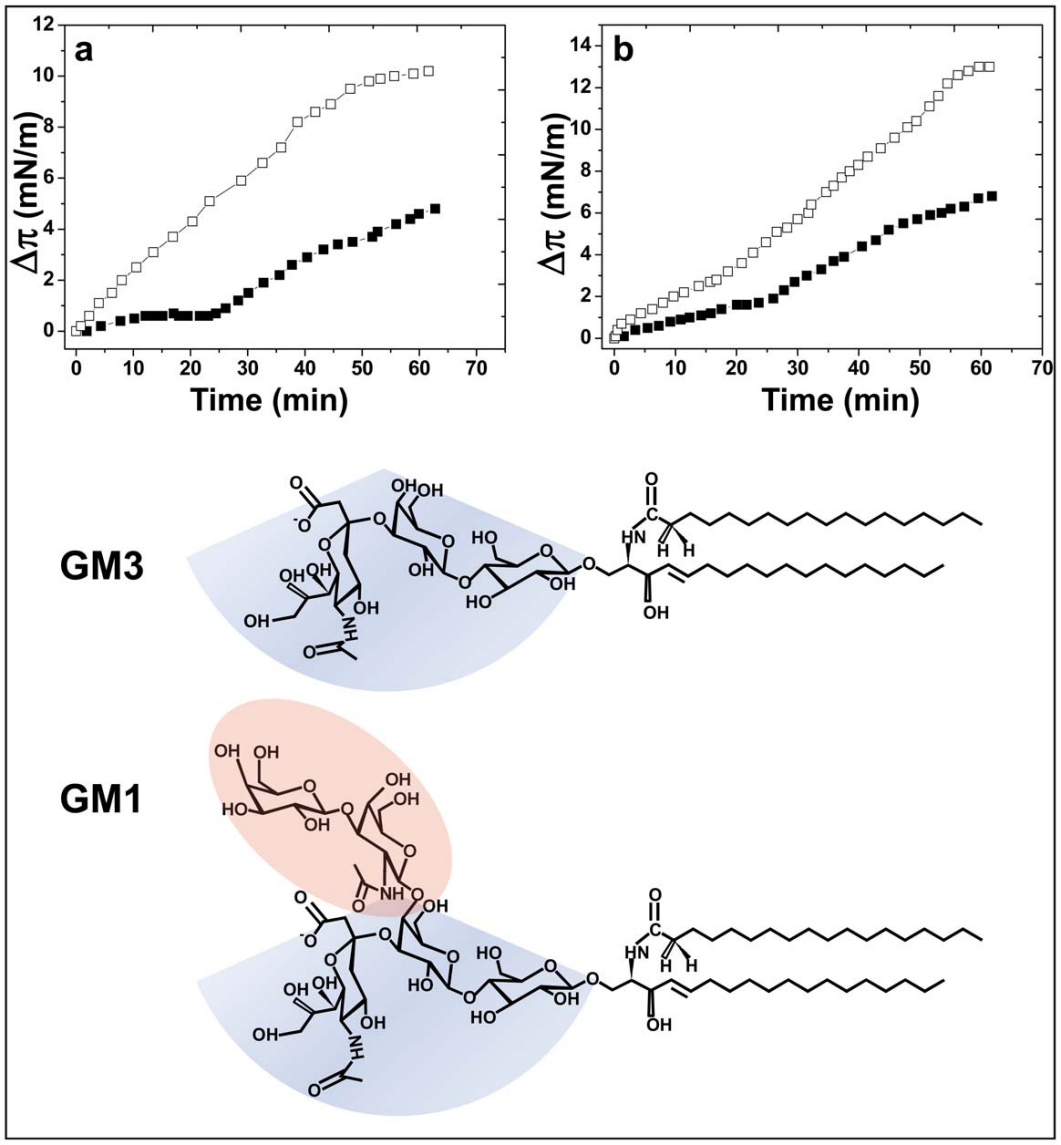
Effects of cholesterol on the recognition of gangliosides GM3 and GM1 by Aβ_1–40_. Kinetics of Aβ_1–40_ insertion into a monolayer of GM3 (**a**) or GM1(**b**) in either the absence (▪) or presence of cholesterol (□). The pure ganglioside and mixed ganglioside/cholesterol (1∶1, mol:mol) monolayers were prepared at an initial surface pressure of 13–15 mN.m^−1^ as indicated in Materials and Methods. The data show the evolution of the surface pressure following the injection of Aβ_1–40_ (1 µM) in the aqueous phase underneath the monolayer. Each experiment was performed in triplicate and one representative curve is shown (S.D. <10%). The chemical structures of GM3 and GM1 (with a C18:0 fatty acid) are shown in the lower panel. Note that the chemical structure of the glycone moiety of GM1 is derived from the one of GM3 (GM3, *NeuAcα2-3Galβ1-4Glcβ1-Cer*; GM1, *Galα1-3GalNacβ1-4-* (highlighted in red) branched on the second Gal residue of GM3. The common oligosaccharide part shared by GM3 and GM1 is highlighted in blue.

Since gangliosides are known to bind to Alzheimer's amyloid peptides and to affect their aggregation [12], [13], [32], [33], we studied the effect of cholesterol on the interaction between Aβ_1–40_ and the monosialylated gangliosides GM3 and GM1 (**Figure 3**). These gangliosides, which were purified from bovine brain (GM1) or buttermilk (GM3), contained only NFA species (see **Table 1** for fatty acid content and **Figure 3** for chemical structures). In absence of cholesterol, both gangliosides were recognized by Aβ_1–40_ as shown by the kinetics of surface pressure increase after injection of Aβ_1–40_ underneath a monolayer of GM3 (**Figure 3a**) or GM1 (**Figure 3b**). However, the values of Δπ_max_ were of low magnitude (**Table 2**), which indicated a moderate affinity of the peptide for these gangliosides compared with GalCer-HFA. As for GalCer-NFA and GalCer-C12, cholesterol had a stimulatory effect on Aβ_1–40_ insertion within GM3 (**Figure 3a**) and, although to a lesser extent, GM1 monolayers (**Figure 3b**). Namely, cholesterol induced a 2.5-fold increase for GM3 and a 1.55-fold increase for GM1, as assessed by comparing the Δπ_max_ values. A detailed analysis of the kinetic parameters of these interactions is presented in **Table 2**.

Overall, these data indicated that cholesterol reproducibly facilitates the interaction of Aβ_1–40_ with all the GSLs that have a non-hydroxylated fatty acid. This effect was reproducibly observed with various NFA-GSLs (GalCer-NFA, GalCer-C12, LacCer-C8, GM3, and GM1) containing biochemically distinct fatty acids (Table 1). On the opposite, cholesterol decreases the interaction of Aβ_1–40_ with GalCer-HFA. Thus the OH group linked to the C2 of the fatty acid seems to play a critical role for Aβ_1–40_ recognition by GSLs, and cholesterol probably acts at this level. To unravel the role of cholesterol on GSL conformation in the context of fatty acid hydroxylation, we performed a series of molecular modeling simulations.

### Molecular Modeling Simulations of Cholesterol-GSL Interactions

**Figure 4:**
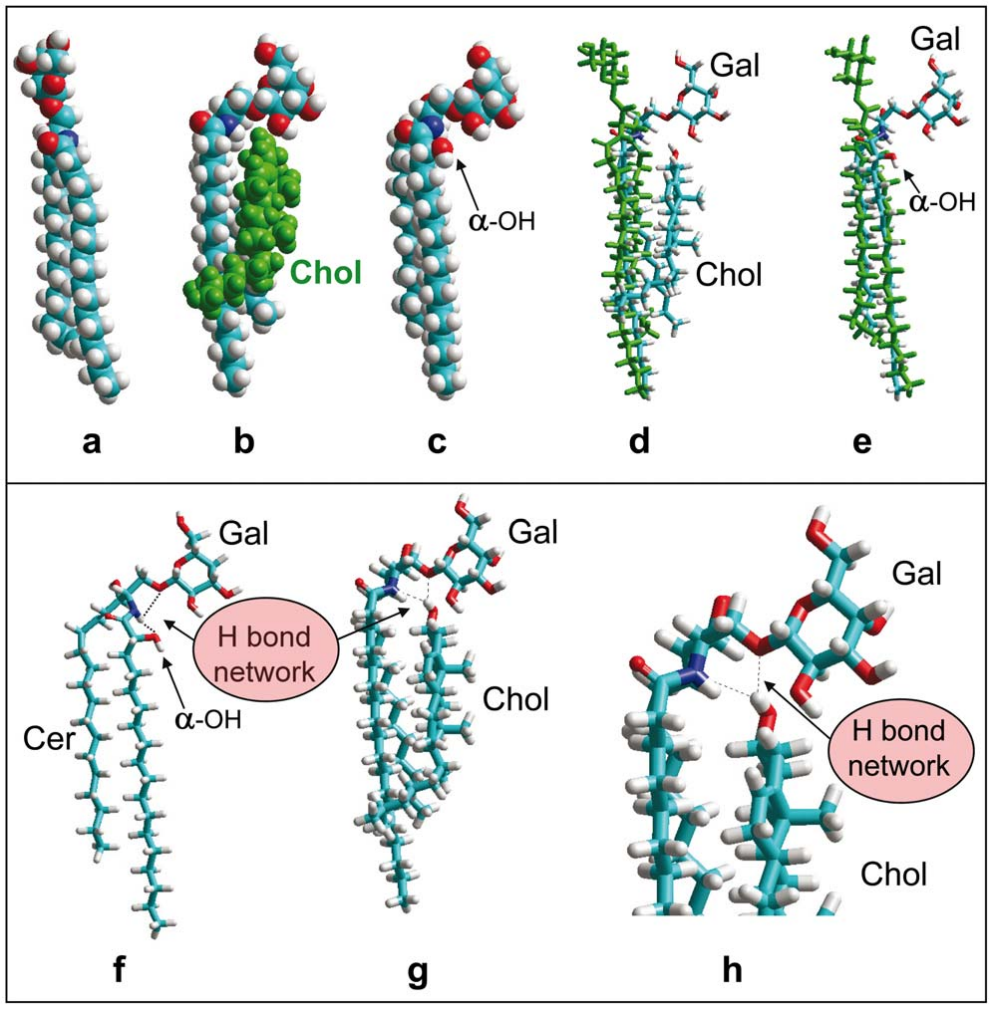
Molecular modeling simulations of GalCer-HFA and GalCer-NFA (alone or complexed with cholesterol). Molecular dynamics simulations were performed as indicated in Materials and Methods. **a-** GalCer-NFA; **b-** GalCer-NFA complexed with cholesterol (in green); **c-** GalCer-HFA (the α-OH group is indicated); **d-** Superposition of GalCer-NFA (identical to **a**, colored green) with the GalCer-NFA/cholesterol complex (identical to **b**). Note the distinct orientation of the galactose headgroup (Gal); **e-** Superposition of GalCer-NFA (in green) with GalCer-HFA; **f-** In GalCer-HFA, the galactose head group is maintained in a shovel-like conformation by a network of H-bonds involving the NH of sphingosine (donor group) and the 2-OH of the fatty acid and the oxygen atom of the glycosidic bond (both acceptor groups); **g-** In GalCer-NFA complexed with cholesterol, the galactose headgroup is also maintained in a typical shovel-like conformation through a network of H-bonds involving the OH of cholesterol (donor group), the NH of sphingosine and the oxygen atom of the glycosidic bond (both acceptor groups). **h-** Higher magnification of the H-bond network in GalCer-NFA complexed with cholesterol.

X-ray diffraction studies have revealed that the galactose ring of GalCer-NFA protrudes at 180° with respect to the main axis of the ceramide backbone [27]. Molecular dynamics studies of GalCer-NFA remarkably converged to the same type of conformation (**Figure 4a**). A remarkable fit between GalCer-NFA and cholesterol could be found (**Figure 4b**), the complex being stabilized by both van der Waals forces and H-bonds. In particular, a H-bond network involving i) the OH group of cholesterol, ii) the NH of sphingosine, and iii) the oxygen atom of the glycosidic bond, could be predicted (**Figure 4g–h**). To better illustrate the effect of cholesterol on the conformation of GalCer-NFA, the GSL alone (in green) has been superimposed on the structure of the GalCer-NFA/cholesterol complex (**Figure 4d–e**).

In GalCer-HFA, the 2-OH group restricts the conformation of the galactose ring so that the molecule adopts a typical L-shape structure (**Figure 4c**). This is in full agreement with crystallographic data which demonstrated that the orientation of the galactose ring in GalCer-HFA is constrained by a network of H-bonds involving i) the 2-OH group of the acyl chain, ii) the NH of sphingosine, and iii) the oxygen atom of the glycosidic bond [26], [27]. This H-bond network is shown in **Figure 4f** which shows the original structure of GalCer-HFA deduced from X-ray diffraction studies [26]. Overall, these data strongly suggest that the 2-OH group of the acyl chain and cholesterol have a comparable conformational effect on GalCer. The molecular mechanism is similar but a detailed analysis shows that in the case of GalCer-HFA, the 2-OH group is a H-bond acceptor (**Figure 4f**) whereas the OH group of cholesterol is a H-bond donor (**Figure 4g–h**). Yet in both cases, the OH group reinforces the strength of the H-bond between the NH group of sphingosine and the oxygen atom of the glycosidic bond, thereby forcing the GSL to adopt an L-shape structure.

### Molecular Modeling Simulations of Aβ_1–40_-GSL Interactions

**Figure 5:**
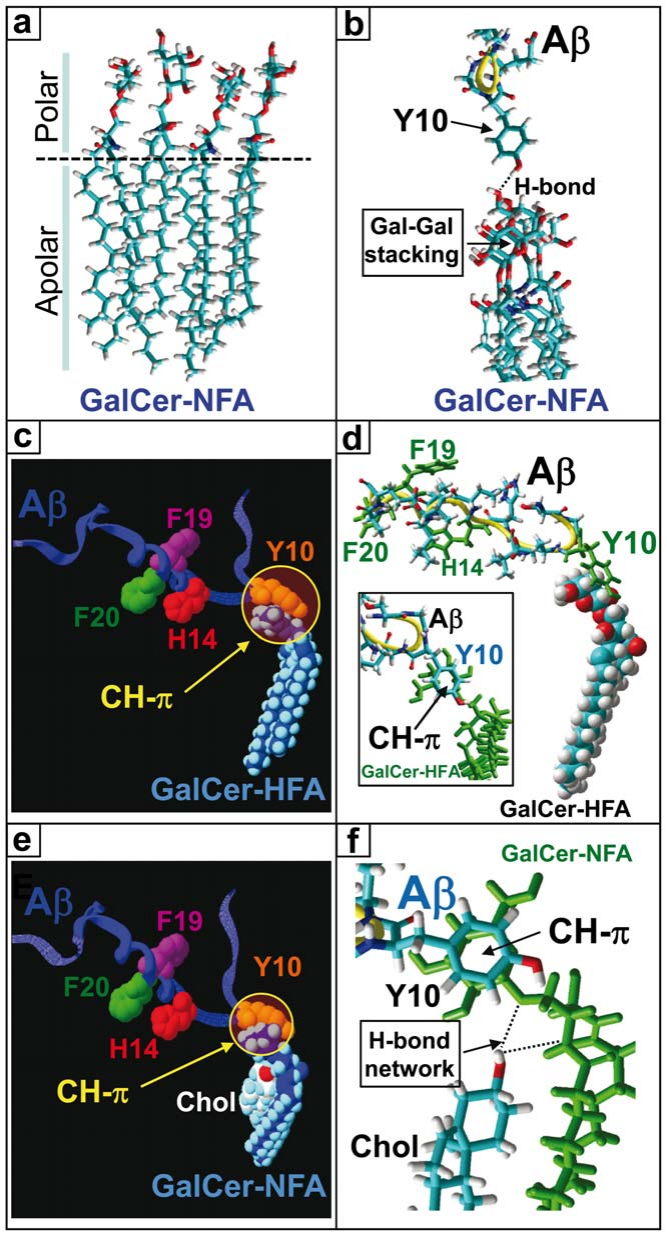
Sugar-aromatic CH-π stacking interactions between Aβ_1–40_ and GalCer-HFA or GalCer-NFA complexed with cholesterol. Molecular dynamics simulations were performed as indicated in Materials and Methods. **a-** View of a cluster of 4 GalCer-NFA molecules in a plasma-membrane compatible orientation. Interactions in both the polar and apolar parts of the GSL stabilize the complex. The dotted line indicates the polar-apolar interface. **b-** The same cluster of GalCer-NFA molecules shown in (**A**) has been merged with Aβ_1–40_ (PDB entry # 1BA6) to search for potential interactions between Aβ and GalCer-NFA. No obvious fit could be found, the only predicted interaction being the H-bond between the phenolic OH group of Y10 and the CH_2_OH group of one of the galactose rings. Note that the aromatic side chain of Y10 cannot stack onto any galactose ring, none being accessible. The stacking of the galactose headgroups of vicinal GalCer-NFA molecules (Gal-Gal stacking) is indicated by an arrow. **c-** CH-π stacking interaction between the galactose ring of GalCer-HFA and the aromatic side chain of the Y10 residue in Aβ_1–40_. Note that the peculiar geometry of the complex leaves residues H14 and F20 accesible for complementary interactions with membrane lipids, whereas residue F19 is rejected on the opposite side. **d-** Detailed view of the complex between GalCer-HFA and Aβ (to improve clarity only residues 8–22 of Aβ_1–40_ are shown). The perfect geometry of the CH-π stacking interaction between the galactose ring and the aromatic side chain of Y10 is illustrated in the inset. **e-** In presence of cholesterol (Chol), the galactose headgroup of GalCer-NFAadopts a specific conformation which renders the galactose ring accessible for a CH-π stacking interaction with the Y10 residue of Aβ_1–40_. **f-** Detailed view of the complex between cholesterol, GalCer-NFA, and Aβ (residues 8–22 of Aβ_1–40_). The CH-π stacking interaction and the H-bond network stabilizing the active conformation of GalCer-NFA are shown.

Our physicochemical studies (**Figures 1** **–** [Fig pone-0009079-g002] **3**) suggested that only the OH-constrained L-shape structure of GalCer is recognized by Aβ_1–40_. So we performed a series of molecular modeling simulations to investigate the possibilities of interaction between the amyloid peptide and each type of GalCer (**Figure 5**). In these experiments, we worked with the structure of Aβ_1–40_ which has been obtained by NMR studies of micellar systems [30]. We did not find any significant fit for Aβ_1–40_ and a single molecule of GalCer-NFA. We observed that individual GalCer-NFA molecules could readily form a patched structure, each galactose ring stacked onto its neighbours (**Figure 5a–b**). Even in this case, there was no obvious fit between these GalCer-NFA molecules and the peptide. This suggests that the peptide and the GSL do not have any geometric nor chemically compatible domains. Indeed, the only possible mode of interaction between Aβ and a cluster of GalCer-NFA was a single H-bond which left the aromatic side chain of residue Y10 fully exposed to the solvent (**Figure 5b**).

In contrast, the planar apolar surface of galactose in GalCer-HFA [31] allowed the establishment of a coordinated CH-π stacking interaction with the aromatic side chain of the Y10 residue (**Figure 5c–d**). As a consequence, the whole structure of Aβ_1–40_ spread onto the surface of the reconstituted GalCer-HFA membrane. In this orientation, residues H14 and F20 are facing the membrane, whereas residue F19 is rejected on the opposite side. A similar interaction could also be predicted between the conformer of GalCer-NFA constrained by cholesterol and the amyloid peptide, through aromatic stacking with Y10 (**Figure 5e–f**). Overall these data suggests that both the 2-OH group of GalCer-HFA and, in the case of GalCer-NFA, the OH group of vicinal cholesterol, orient the sugar head of GalCer in such a way that it becomes compatible with the establishment of CH-π stacking interactions with aromatic amino acid side chains.

### Physicochemical Experiments in Support of Molecular Modeling Data

**Figure 6:**
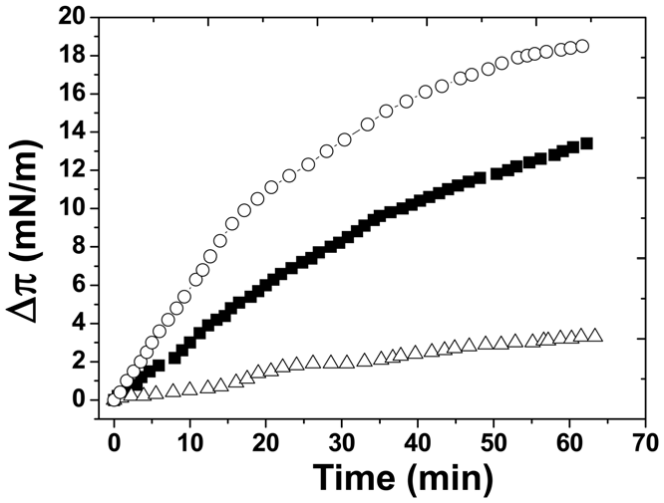
Effects of sodium salts on the interaction between GalCer-HFA and Aβ_1–40_. Monolayers of GalCer-HFA were prepared at an initial surface pressure of 13–15 mN.m^−1^ on a subphase of pure water (▪), 0.1 M NaCl (○) or 0.1 M NaF (▵). The data show the evolution of the surface pressure following the injection of Aβ_1–40_ (1 µM) in the subphase. Each experiment was performed in triplicate and one representative curve is shown.

**Figure 7:**
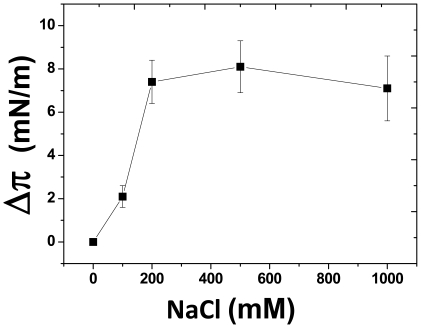
Change in surface pressure as a function of time upon injection of Aβ_1–40_ into a water subphase containing various NaCl concentrations. The concentration of Aβ_1–40_ in the subphase is 5 µM and the subphase temperature is 20°C. The results are expressed as the mean ± S.D. of three independent surface pressure measurements performed after 65 min of incubation with the peptide.

Several experiments were performed in order to confirm the interpretations based on the molecular modeling studies. Firstly, we used NaF (from 0.1 to 1M) as a breaker of H bonds, this effect being due to the high electronegativity of the fluor atom [34]. In presence of NaF, there was a marked inhibition of both v_i_ and Δπ_max_ in the monolayer assay, showing that destabilizing the hydrogen bond network decreased the affinity of Aβ for GalCer-HFA (**Figure 6** and **Table 2**). Secondly, we studied the effect of high concentrations of NaCl (from 0.1 to 1M) on the interaction between Aβ_1–40_ and GalCer-HFA. By increasing the ionic strength, this salt destabilizes electrostatic interactions whereas it reinforces hydrophobic interactions. As shown in **Figure 6** and **Table 2**, the presence of NaCl in the subphase resulted in a marked increase in Aβ_1–40_-GalCer-HFA association, as assessed by both the values of v_i_ and Δπ_max_. These data suggested that hydrophobic forces, rather than electrostatic interactions, are involved in the stabilization of the peptide-GSL complex. This is consistent with the hydrophobic stacking interaction between the galactose ring of GalCer and the aromatic side chain of Y10 in Aβ (**Figure 5**). However, the stimulatory effect of NaCl could also be interpreted as a salting out effect of the peptide, i.e. an increased concentration of the peptide at the interface which could change the surface pressure independently of the association between Aβ and GalCer-HFA. To investigate this possibility, we studied the effect of various NaCl concentrations on the spontaneous tensioactivity of Aβ_1–40_. In this experiment Aβ_1–40_ (5 µM, i.e. 5 times the concentration used to study Aβ-GSL interactions) was injected in the water phase containing increasing concentrations of NaCl (ranging from 100 to 1,000 mM). No lipid was spread at the interface so that any change in the surface pressure reflected exclusively the interfacial recruitment of Aβ_1–40_. The surface pressure increase measured after 65 min of incubation was plotted against the NaCl concentration (**Figure 7**). A maximal value of 8.1 mN.m^−1^ was obtained between 200 and 1,000 mM NaCl. At 100 mM NaCl, Aβ_1–40_ increased the surface pressure by only 2.1 mN.m^−1^ (to be compared with the data of **Figure 6**). Thus, although the above-mentioned salting out effect did exist, it could not, by itself, account for the stimulatory effect of NaCl on the association between Aβ_1–40_ and GalCer-HFA. Overall these physicochemical data were in good agreement with the predictions of our molecular modeling studies, which underscored the role of hydrophobic stacking aromatic interactions (reinforced by NaCl), and H-bonds (destabilized by NaF) in Aβ-GalCer interactions.

## Discussion

Although it is widely admitted that cholesterol and GSLs play a pivotal role in Aβ release, conformation, oligomerization, and fibrillogenesis [16], the molecular mechanisms controlling the complex interplay between Aβ peptides and these lipids are not clear. In particular, the biochemical diversity of GSLs in their ceramide moiety, especially the hydroxylation status of the C2 in the acyl chain, has not been appreciated in Aβ-GSLs binding studies. We believe that this issue is critical for at least three reasons: i) quantitatively, NFA and HFA species of the same GSL can be expressed in roughly similar amounts in brain tissues, as it is the case for GalCer in myelin [35]; ii) the 2-OH group has a major impact of the conformation of the GSL, due to intramolecular H-bonding possibilities [36]; iii) gender-specific expression of NFA vs. HFA ceramides have been observed in a mouse model of Alzheimer's disease, and this biochemical feature could be related to the increased propensity of women to develop Alzheimer's disease [37].

Using the Langmuir monolayer technique [6], we studied the interaction between Aβ_1–40_ and various HFA- and NFA-GSLs with known fatty acid composition. We showed that Aβ_1–40_ interacted more efficiently with a pure monolayer of GalCer-HFA than with GalCer-NFA, as assessed by both the initial velocity of the insertion reaction and the increase in the surface pressure induced by the peptide (**Figure 1**). Similar results were previously obtained with HIV-1 gp120, a protein sharing with Aβ peptides a common sphingolipid-binding domain (SBD) involved in GalCer recognition [10]. On the basis of these data, Hebbar *et al.* [11] have designed a fluorescent SBD probe targeting lipid raft domains on live cells. This probe, derived from the part of Aβ containing the SBD we have defined [10], [38], recognized a mixture of bovine brain GalCer containing both GalCer-HFA and GalCer-NFA, but not synthetic GalCer-NFA. Therefore, our data are in perfect agreement with those of Hebbar *et al.* [11].

In the present study, the interaction of Aβ_1–40_ with lipid monolayers has been performed at a surface pressure (13–15 mN.m^−1^) that is lower than the value of 30 mN.m^−1^ currently considered as representative of natural membrane bilayers [39]. The rationale for working at 13–15 mN.m^−1^ is that at these pressures, Aβ_1–40_ does not penetrate into pure cholesterol monolayers (**Table 2**). This allows a clear-cut assessment of the effect of cholesterol on the interaction of Aβ_1–40_ with various natural and synthetic GSL species. Previous data indicated that Aβ_1–40_ readily interacts with a monolayer of GalCer-HFA with an initial surface pressure of 30 mN.m^−1^ [10]. In contrast, under the same conditions, the peptide showed very little interaction with GalCer-NFA and GalCer-C12 monolayers (data not shown). Therefore, one of the most important findings of the present study (strong interaction of Aβ_1–40_ with GalCer-HFA monolayers, weak interaction with GalCer-NFA and GalCer-C12) was confirmed at high surface pressure values that are representative for natural biomembranes.

The other major finding of this study is that cholesterol could transform the inactive GalCer-NFA into a fully active GSL-cholesterol complex recognized by Aβ_1–40_. This stimulatory effect of cholesterol was observed with both natural and synthetic GalCer-NFA (**Table 2**). Control experiments with cholesterol alone and with PS:cholesterol mixed monolayers confirmed that the peptide specifically recognized GalCer-NFA complexed with cholesterol (**Table 2**). The previously established 3D structures of GalCer-NFA and GalCer-HFA [26] immediately suggests a molecular mechanism accounting for these physicochemical data. In GalCer-HFA, the galactose ring is parallel to the membrane, consistent with the establishment of a typical CH-π stacking interaction [40] between the apolar face of galactose and an aromatic side chain of the peptide [29]. This type of interaction has been evidenced for various sugar-protein complexes, including sugar-lectin and GSL-peptide systems [41]. Structure similarity searches combined with various physicochemical approaches suggested that the interaction between GalCer-HFA and Aβ involved the aromatic side chain of Y10 [10]. Interestingly Hebbar *et al.* [11] who showed that a double mutant Aβ peptide (R5A/Y10A) did not bind to GalCer. Altogether, these data are in line with the molecular modeling study of **Figure 5**, in which we assigned a major role for Y10 in GalCer recognition. We conclude that the binding of Aβ to GalCer requires that the galactose ring of the GSL is in a parallel orientation with respect to the membrane, the GSL adopting a typical L-shape. This conformation can be stabilized by an intramolecular H-bond network involving the 2-OH group of GalCer-HFA. In absence of this H-bond network, GalCer-NFA cannot adopt this active conformation (**Figures 4** **–** **5**). The galactose rings are not accessible to the Y10 residue, and there is no possibility for a stable interaction with Aβ. Yet cholesterol can transform this inactive conformation by forcing the galactose ring to adopt a parallel orientation compatible with the stacking of Y10. As expected, this hydrophobic CH-π stacking interaction was reinforced in presence of high concentrations of NaCl in the subphase underneath the monolayer (**Figure 6** **and** **Table 2**). Finally, destabilizing the intramolecular H-bond network with NaF strongly impaired the insertion of Aβ, which strongly supported the conclusions drawn on the basis of our modeling studies. In total agreement with our data, Ikeda & Matsuzaki [42] also identified H-bonding and hydrophobic interactions as the driving forces responsible for Aβ_1–40_-glycolipid interactions in lipid bilayer systems.

The specific effect of cholesterol on GSL conformation is not necessarily related to the condensing activity of the sterol on GSL monolayers. Indeed, comparative studies of mixed monolayers suggested that at 22°C, cholesterol displays a high condensing effect on dihexosylceramides, but on GalCer monolayers [43]. At 37°C, cholesterol was found to specifically condense GalCer-NFA, but not GalCer-HFA [24]. Thus our data cannot be interpreted in terms of cholesterol-induced condensation of GSL species, but definitely in terms of GSL conformation, which can be finely tuned by vicinal cholesterol molecules. That the incorporation of cholesterol in a GalCer-NFA monolayer could modulate the average orientation of the sphingolipid is in line with the subsequent studies of GalCer-cholesterol interactions conducted by Smaby et al. [44].

Finally, the regulatory activity of cholesterol on the conformation of GSL and the resulting impact on Aβ recognition was also observed for more complex GSLs such as LacCer, GM3 and GM1. This suggests that cholesterol exerts a wide regulatory activity on Aβ-GSL interactions, whose direction depends on the hydroxylation status of the fatty acid chain of the GSL: stimulation of Aβ binding for NFA-GSLs, inhibition of Aβ binding for HFA-GSLs. That “variations in GSL fatty acid composition may mediate aglycone regulation of GSL membrane receptor function by a differential interaction with cholesterol and other membrane components” has been recently discussed by Lingwood *et al.* [45]. As stated above, fatty acid hydroxylation of GSLs is generally associated with improved ligand binding [11], [28], [29], [46]–[48], and this effect has been correlated with the conformation of the sugar head group of GSLs based on the crystallographic studies published by Pascher and co-workers [26], [27]. In line with all these studies, our data demonstrate that the ceramide part of GSLs is critical for Aβ binding, and that cholesterol can, for those GSLs which are normally not recognized by Aβ (i.e. NFA-GSLs), forces them to adopt a conformation compatible with Aβ. This fine tuning of GSL conformation may be highly relevant to the neuropathology of Alzheimer's disease.
